# Single operator experience of endoscopic submucosal dissection for Barrett's neoplasia in a North American academic center

**DOI:** 10.1002/deo2.81

**Published:** 2021-12-14

**Authors:** Douglas Motomura, Robert Bechara

**Affiliations:** ^1^ Department of Gastroenterology, Kingston Health Sciences Center Kingston Canada

**Keywords:** Barrett's esophagus, endoscopic gastrointestinal surgery, endoscopic submucosal dissection, esophageal neoplasms, gastrointestinal endoscopes

## Abstract

**Objectives:**

Endoscopic submucosal dissection (ESD) is carving out an increasing role in the treatment of Barrett's associated neoplasia. ESD provides the advantage of en‐bloc resections and greater R0 resection rates. We aim to present outcomes from one of the largest single‐center cohorts of esophageal ESD in North America.

**Methods:**

All patients undergoing esophageal ESD for Barrett's neoplasia between Oct 2016 and June 2020 at a Canadian tertiary care center were included. Demographic, procedural data, and lesion characteristics are presented. Subgroup analysis was performed on patients who underwent extensive resection (≥75% of esophageal circumference) and the patients who developed strictures.

**Results:**

Thirty‐four patients were included in the series. The median lesion diameter was 5.7 cm and the median procedure time was 129 min. The en‐bloc resection rate was 97%, and the R0 resection rate was 91%. Curative resection was achieved in 82% of patients. Upstaging in histology occurred in 59% of cases. Two adverse events occurred, and there were no perforations. Procedural outcomes were similar in patients with extensive resections, but those with ≥75% circumferential resection developed more strictures (65% vs. 6.3%, *p* < 0.01). Stricture formation was associated with extensive resection (odds ratio [OR]: 27.5, *p* < 0.01) and longer lesion diameter (OR: 1.7, *p* = 0.02).

**Conclusion:**

Our experience with ESD for Barrett's related neoplasia shows excellent en‐bloc and R0 resection rate, and provides more accurate histological specimens. Curative resection is possible in the majority of cases, including those with extensive resections. Further investigation into stricture prophylaxis will be useful as near circumferential resections are attempted.

## BACKGROUND

Endoscopic submucosal dissection (ESD) is an endoscopic technique that allows for en‐bloc resection of larger lesions to facilitate pathologic examination. Esophageal ESD was first performed in the esophagus in Japan for the treatment of squamous cell carcinoma (SCC).^1^ However, the western landscape of esophageal neoplasia differs significantly. In contrast to the Asia and Oceanic regions, the majority of esophageal cancers encountered in North America are Barrett's associated esophageal adenocarcinoma (EAC).^2^

Barrett's esophagus (BE) is a premalignant condition involving a metaplastic change of the normal squamous esophageal epithelium to an intestinal columnar lining. The rate of development of adenocarcinoma in non‐dysplastic Barrett's is 0.33% annually.^3^ However, with high‐grade dysplasia (HGD), the annual incidence of EAC increases up to 6.5%.^4^ Endoscopic therapy is suggested for Barrett's with low‐grade dysplasia, HGD, intramucosal carcinoma (T1a‐EAC), and consideration for select superficial submucosal lesions (T1b‐EAC).^5^

Although this is classically performed with endoscopic mucosal resection (EMR) of visible lesions, followed by eradication of flat Barrett's with radiofrequency ablation, there are several limitations. Larger lesions resected via EMR are removed piecemeal. The use of ESD for en‐bloc resection can provide more accurate histologic specimens, and has been shown to increase R0 resection rates.^6^

We aim to present our results with esophageal ESD, focusing on a large cohort of ESD for BE.

## MATERIALS AND METHODS

### Patient selection

This is a retrospective review of a prospectively maintained de‐identified database. Consecutive patients undergoing esophageal ESD were included between Oct 2016 and June 2020 at a single tertiary academic center in Kingston, Ontario, Canada. Inclusion criteria for ESD were patients who had BE and suspected early adenocarcinoma (intramucosal or superficial submucosal) or extensive HGD. There were no pre‐specified exclusion criteria from the cohort, but patients who were suspected or proven to have deep submucosal involvement or T2 disease did not undergo ESD. Local ethics board approval was obtained for the study. Patient consent is obtained at the time of procedure and includes the usage of de‐identified information for research purposes.

### Data collection

Demographic information was available on all patients, including age, ASA score, sex, initial histologic diagnoses from biopsies, and previous treatment. Procedural characteristics such as procedure time, efficiency, lesion size, and lesion area were recorded. Efficiency in this study is defined as the procedural time (min) per area resected (cm^2^). Histological features are presented. Depth of invasion is measured in micrometers and categorized according to the depth of submucosa involved (sm1–3).^7^ Superficial (Sm1) was therefore defined as an invasion into the submucosa of 500 μm or less. Subgroups categorized by stricture formation were analyzed.

Our primary outcome of interest is R0 resection rates, defined as negative deep and lateral histologic margins of the highest‐grade lesion within the target area. Secondary outcomes of interest include curative resection rates, differences between initial and final pathology, and adverse events. Curative resections are defined as R0 resections lacking other high‐risk features such as poor differentiation, histologic lymphovascular invasion, or deep submucosal involvement. We performed subgroup analysis on a cohort of patients with extensive (≥75% of esophageal circumference) resections, as well as a cohort of patients who developed esophageal strictures following the procedure, which in this series were defined as luminal narrowing noted on follow up endoscopy requiring endoscopic treatment.

### Procedural technique

All cases were performed by one experienced operator (Robert Bechara). Robert Bechara has completed a formal year‐long fellowship at Showa University, Tokyo, Japan, focusing on POEM and ESD procedures. Procedures were mostly performed under general anesthesia and endotracheal intubation, however, a select few cases used conscious sedation only. DualKnife (KD‐650; Olympus) or DualKnife J (KD‐655; Olympus) were used for the procedures. Antithrombotic agents were held in general accordance with American Society for Gastrointestinal Endoscopy recommendations.^8^ The lesions were identified with a combination of white‐light and narrow‐band imaging, and the usage of acetic acid when applicable. The margins were marked using soft coagulation and the closed tip of the Dualknife J. After marking, a submucosal injection was performed, mucosal incision was made, and careful dissection of the submucosa was completed using saline and indigo carmine solution for submucosal injection. After complete resection and retrieval, the specimens were pinned down and measured, then fixed in formalin for histopathologic examination. Following the procedure, patients with extensive resections (defined as those with circumferential resection of 75% or more) were given individualized plans for stricture prophylaxis that included a combination of PPI, sucralfate, topical swallowed steroids, and systemic steroids (if no contraindication). If patients had residual flat BE following ESD, complete eradication was typically performed with EMR or ablation after appropriate healing was achieved.

### Statistics

Descriptive results are presented as the median and interquartile range (IQR). Categorical variables are expressed as percentages. Subgroup data were compared with Mood's median test for continuous data, and Pearson's chi‐square test for categorical data. Subgroup analysis was performed comparing patients with stricturing and those without. Univariable binary logistic regression was performed. Data are presented as odds ratio (OR) with 95% confidence interval (CI). Data analysis was performed with Microsoft Excel with the Data Analysis add‐in and SPSS Statistics (Version 26, SPSS, Chicago, Ill).

## RESULTS

Thirty‐four patients underwent esophageal ESD during the study period. Demographic data are presented in Table 1. The median age was 67, and 85% of patients were male. The majority of patients had HGD on pre‐resection biopsy (18 patients, 53%) followed by T1a‐EAC (10 patients, 29%). Two patients did not have dysplasia on their index biopsies, but endoscopic examination harbored features suspicious for at least HGD (irregular microvascular pattern, demarcation line, and early loss of acetowhitening) over long segments. Eleven patients (32%) had prior treatment, which includes EMR, ESD, and radiation therapy. The majority of patients had a long segment (≥3 cm) Barrett's Esophagus (85%). Approximately half (47%) of the lesions were flat (Paris 0‐IIb), with the rest comprising of mixed morphologies, raised or sessile lesions.

**TABLE 1:** Patient and lesion characteristics

Demographics	(*N* = 34)
Sex (M)	29 (85.3%)
Age (Median, IQR)	67 (62–73)
ASA	
1	2 (5.9%)
2	10 (29.4%)
3	22 (64.7%)
Baseline pathology	
No dysplasia	2 (5.9%)
Low‐grade dysplasia	1 (2.9%)
High‐grade dysplasia	18 (52.9%)
Intramucosal carcinoma	10 (29.4%)
Deep invasion	2 (5.9%)
No prior biopsy	1 (2.9%)
Prior treatment	11 (32.4%)
Macroscopic type (Paris)	
0‐I	6 (17.6%)
0‐IIa	4 (11.8%)
0‐IIb	16 (47.1%)
0‐IIc	0
0‐III	0
Mixed	8 (23.5%)
Length of BE	
Short segment (<3cm)	5 (14.7%)
Long segment (≥3cm)	29 (85.3%)

Abbreviation: ASA, American Society of Anesthesiologists.

Technical success was high, with 33 patients having a successful en‐bloc resection (97%, Table 2). One case was aborted due to encountering multiple varices. There were no piecemeal resections. R0 resection was completed in 31 patients (91%). Despite R0 resection, three of these patients were considered to have non‐curative resections. Two patients with R1 resections had positive deep margins. One patient was suspected to have deep invasion endoscopically during the procedure, and partial resection of the circular smooth muscle confirmed the involvement of the muscularis propria. The second patient had lymphovascular involvement with poor differentiation on histology and underwent neoadjuvant chemoradiation and esophagectomy which demonstrated patchy foci in the muscularis propria.

**TABLE 2:** Procedural characteristics

Procedural outcomes	*N* = 34
En bloc resection	33 (97.1%)
R0 resection	31 (91.1%)
Curative resection	28 (82.4%)
Max diameter (cm, median, IQR)	5.7 (4.2–7.5)
Area (cm^2^)	17.8 (9.0–28.3)
Procedure time (min)	129 (66–200)
Efficiency (min/cm^2^)	6 (4–10)
% Circumferential	
<25%	0 (0%)
25%–49%	10 (30.3%)
50%–74%	6 (18.2%)
75%–100%	17 (51.5%)
Adverse events	
Perforation	0 (0%)
Aspiration	1 (2.9%)
Delayed bleeding	1 (2.9%)
Stricture Formation	12 (35.3%)

Seventeen patients (52%) had ≥75% of the esophageal circumference resected, and eight (24%) had 100% circumferential resections. There were two adverse events (6%) encountered in the cohort. One patient had an aspiration event on extubation in the operating room care and required re‐intubation. The second patient developed bleeding after discharge and returned on post‐procedure day 4. Conservative management was sufficient, and no‐repeat endoscopy or blood transfusions were required. A minority (33%) of patients were admitted, with eight patients admitted prophylactically after a 100% circumferential resection, with the remainder admitted for post‐operative pain control. The median hospital stay in this group was 1 day. A significant minority of patients required dilations at follow‐up endoscopies (12 patients, 36%), but there were no refractory strictures. A median of three balloon dilations were required in patients who developed strictures (range: 1–19)

Final pathology revealed adenocarcinoma in the majority of procedures (70%, Table 3). Of the carcinomas found on pathology, the majority were T1a (65%). Over half of the carcinomas were well‐differentiated (57%) with three patients (13%) having a component of poor differentiation. Four patients (12.1%) had high‐risk histological features which included lymphovascular invasion, perineural invasion, and tumor budding. The majority of cases were upstaged from the biopsy specimen to the final pathology (59%, Table 4)

**TABLE 3:** Histologic outcomes

Final pathology (n = 33)	
LGD	2 (6.1%)
HGD	8 (24.2%)
Adenocarcinoma	23 (69.7%)
CA depth (*n* = 23)	
M	15 (65.2%)
Sm1	4 (17.4%)
Sm2	1 (4.3%)
Sm3	1 (4.3%)
T2+	2 (8.7%)
Differentiation (*n* = 23)	
Well	13 (56.5%)
Moderate	7 (30.4%)
Poor	3 (13.0%)
Histologic features	
Lymphovascular invasion (*n* = 23)	3 (13.0%)
Perineural invasion (*n* = 23)	0 (0%)
Tumor budding (*n* = 22)	1 (4.5%)

Abbreviations: CA, cancer; HGD, high‐grade dysplasia; IMCA, intramucosal carcinoma; LGD, low‐grade dysplasia; Sm, submucosa.

**TABLE 4:** Comparison between pre‐ESD biopsy and post ESD histology

	**Post ESD pathology**							
**Biopsy**	**No dysplasia**	**LGD**	**HGD**	**IMCA**	**Sm+**	**Change in diagnosis**		
No dysplasia	0	2	0	0	0	Downstaged	3	(9.4%)
LGD	0	0	1	0	0	Properly Staged	10	(31.3%)
HGD	0	0	4	11	2	Upstaged	19	(59.4%)
IMCA	0	0	3	4	3			
Sm+	0	0	0	0	2			

Abbreviations: CA, cancer; HGD, high‐grade dysplasia; IMCA, intramucosal carcinoma; LGD, low‐grade dysplasia; Sm, submucosa.

Outcomes between patients with ≥75% circumferential resection and <75% circumferential were compared (Table 5). Patients undergoing more extensive resections were younger (median: 63 years vs. 71.5 years, *p* ≤ 0.01), had larger lesion diameter (7.1 cm vs. 4.1 cm, *p* ≤ 0.01), and area (24cm^2^ vs. 8.8 cm^2^, *p* ≤ 0.01). This led to longer procedure times (155 min vs. 96 min, *p* ≤ 0.01). Efficiency was lower in more circumferential lesions, but this did not reach statistical significance (5 min/cm^2^ vs. 7 min/cm^2^, *p* = 0.23). R0 and curative resection rates did not differ between groups. Strictures were significantly more common in the ≥75% circumferential group (64.7% vs. 6.3%, *p* ≤ 0.01)

**TABLE 5:** Comparison between extensive (≥75% of esophageal circumference) and limited resections

	**≥75% Circumferential**		
	**No (*n* = 16)**	**Yes (*n* = 17)**	***p***
Sex (M)	14 (87.5%)	14 (82.4%)	0.68
Age	71.5 (69–79.8)	63 (57–65.5)	<0.01
ASA			0.25
1	0 (0%)	2 (11.8%)	
2	4 (25%)	6 (35.3%)	
3	12 (75%)	9 (52.9%)	
Max Diameter (cm)	4.1 (3.3–5.9)	7.1 (5.7–8.3)	<0.01
Area (cm^2^)	8.8 (6.4–17.2)	24 (17.7–43.4)	<0.01
Time (min)	65.5 (45‐152.3)	155 (108.5–245.5)	0.02
Efficiency (min/cm^2^)	7 (5–13)	5 (3–9.5)	0.23
En bloc	16 (100%)	17 (100%)	n/a
R0	15 (93.8%)	16 (94.1%)	0.97
Curative resection	12 (75%)	16 (94.1%)	0.13
Stricture	1 (6.3%)	11 (64.7%)	<0.01

Abbreviation: ASA, American Society of Anesthesiologists

Subgroup analysis was performed on patients who developed strictures (Table 6). Compared against patients who remained stricture‐free, these patients were younger (OR: 0.85), had larger lesions (OR: 1.66), and had a larger percentage of circumferential resection. Of all patients who developed strictures, 11 (92%) had extensive resections (OR: 27.5). Multivariable analysis was not performed given the relatively small number of events per variable.

**TABLE 6:** Analysis of patients who developed esophageal strictures

	**No stricture (*n* = 21)**	**Stricture (*n* = 12)**	**Univariable OR**	***p***
Male (%)	18 (86%)	10 (83%)	0.83 (0.12–5.85)	0.86
Age (years, median, IQR)	70 (67–79.5)	62 (53–63.8)	0.85 (0.75–0.97)	0.01
Maximum Diameter (cm)	4.6 (3.4–6.2)	7.3 (5.7–8.9)	1.66 (1.10–2.51)	0.02
Area (cm^2^)	10.8 (6.9–25.7)	22.3 (20.3–35.8)	1.02 (0.99–1.05)	0.19
≥75% Circumferential	5 (24%)	11 (92%)	27.5 (2.88– 262.33)	<0.01

Abbreviations: IQR, interquartile range; OR, odds ratio.

## DISCUSSION

The primary outcome of R0 resection in our cohort was 91%. This compares favorably to the published pooled R0 resection rates in a recent meta‐analysis of ESD for BE, which was found to be 75%.^9^ Our secondary outcome of curative resection (85%) was also improved from previously published estimates (65%), partially due to the higher R0 resection rates. En‐bloc resection in our study (97%) was similar to previous meta‐analysis (93%).

Median lesion size in our study (5.7 cm) is larger than any of the studies included in a recent meta‐analysis (mean lesion size: 2.7 cm),^9^ with only a small increase in procedural time (median 129 min vs. mean 108 min in pooled studies). Comparatively, a single European center case series of 36 lesions, with a mean size of 51 mm, had mean procedural times of 191 min.^10^ Additionally, a recently published large multi‐center North American cohort included 93 ESDs, with a median lesion size of 39 mm.^11^ Because of the irregular shape of many lesions, we also presented an estimate of lesion size in two dimensions, as well as a measure of procedural efficiency in min/cm^2^, which may help standardize comparisons between studies with varying lesion dimensions and procedural times.

Lesion size has been proposed as a predictor of R1 (incomplete) resection. The previous systematic review compared R0 resection rates in lesions <25mm to lesions ≥25mm, and found pooled rates of 92% and 85% respectively, primarily in superficial SCC.^12^ However, subsequent multivariate analysis in a large cohort of EAC found only depth of invasion (submucosal involvement) to be predictive of R1 resections.^13^ Of the two patients in our study that had completed procedures but R1 resections, one had a lesion greater than 10 cm and 100% circumferential involvement with areas of poor differentiation, and the other had involvement of the muscularis propria (T2).

Nearly 60% of lesions resected were upstaged on final pathology from initial biopsies, including 11 cases upstaged from HGD to T1a‐EAC (65% of HGD biopsies) and two cases upgraded from HGD to T1b‐EAC (12%). The inaccuracy of forceps biopsies has been demonstrated in gastric cancer, where rates of upstaging on endoscopic resection can be 19% when EMR is performed,^14^ and 49% when ESD was used primarily.^15^ In EAC, this has been shown in previous ESD series, where rates of upstaging were 39%,^16^ comparable to the rates in our study.

Stricturing was a common occurrence in our series, with 35% of all comers developing esophageal stricture. All strictures were managed endoscopically. This is higher than the pooled stricture rate of 12% from the meta‐analysis.^9^ However, our cohort differs in the large proportion of near‐circumferential resections. Previous studies in endoscopic therapy for SCC of the esophagus have shown that resection of ≥75% of the esophagus is associated with increased stricture risk, with the OR of 44.^17^ Indeed, in our univariable analysis, the OR of stricturing in a resection 75% or greater in circumference was 27. In a large cohort of ESD for Barrett's neoplasia in Belgium, stricture rates were described as high as 60%.^18^ Similar to our population, the median circumferential resection in that series was 75% (IQR: 66%–80%). Several preventative measures have been proposed, including intraprocedural injection of steroids into the mucosal defect,^19^, ^20^ post‐procedural oral steroids,^21^ and advanced techniques such as tissue engineering.^22^ The patients in our series received individualized regimens of prophylaxis consisting of PPI, sucralfate, topical/swallowed steroids, and systemic steroids. The safest and most effective prophylactic regimen is an ongoing area of research.

The strengths of this study include the population size. This is one of the largest North American single‐center cohorts of esophageal ESD. It is also the first from Canada, where ESD for early GI malignancies is in the nascent stage. Our population is also unique in the relatively larger lesions and greater circumferential resections compared to the previous series, which presents unique challenges in the management of stricturing. The limitations of the study are intrinsic in the single‐operator, single‐center retrospective design. External generalizability may thus be limited. Selection bias among cases is also possible, though arguments can be made against this by noting the larger, more complicated resections attempted. Long‐term outcomes, including important data on clinical recurrence, are yet to be collected. Although large for a case series on this topic, the relatively small subgroup size precluded multivariable analysis.

Overall, this study demonstrates that ESD for Barrett's associated neoplasia is feasible, safe, and effective in the current North American landscape. The procedure has very high R0 resection rates and low major complication rates and can offer an acceptable alternative to surgical management of larger lesions, even with extensive esophageal involvement.

## FUNDING INFORMATION

None.

## ETHICS STATEMENT

Local ethics board approval was received.

## CONFLICT OF INTEREST

Douglas Motomura has no conflict of interest to declare. Robert Bechara is a consultant for Olympus, Pentax, Medtronic and has received honoraria from Pendopharm.
